# Large-scale analysis of delayed recognition using sleeping beauty and the prince

**DOI:** 10.1007/s41109-021-00389-0

**Published:** 2021-06-30

**Authors:** Takahiro Miura, Kimitaka Asatani, Ichiro Sakata

**Affiliations:** Department of Technology Management for Innovation, School of Engineering, The University of Tokyo, 7-3-1 Faculty of Engineering Bldg 3, Hongo, Bunkyo, Tokyo, 113-8656 Japan

**Keywords:** Bibliometrics, Citation network, Delayed recognition, Sleeping beauty, Prince, Interdisciplinary fusion, Production of knowledge

## Abstract

Delayed recognition in which innovative discoveries are re-evaluated after a long period has significant implications for scientific progress. The quantitative method to detect delayed recognition is described as the pair of Sleeping Beauty (SB) and its Prince (PR), where SB refers to citation bursts and its PR triggers SB’s awakeness calculated based on their citation history. This research provides the methods to extract valid and large SB–PR pairs from a comprehensive Scopus dataset and analyses how PR discovers SB. We prove that the proposed method can extract long-sleep and large-scale SB and its PR best covers the previous multi-disciplinary pairs, which enables to observe delayed recognition. Besides, we show that the high-impact SB–PR pairs extracted by the proposed method are more likely to be located in the same field. This indicates that a hidden SB that your research can awaken may exist closer than you think. On the other hand, although SB–PR pairs are fat-tailed in Beauty Coefficient and more likely to integrate separate fields compared to ordinary citations, it is not possible to predict which citation leads to awake SB using the rarity of citation. There is no easy way to limit the areas where SB–PR pairs occur or detect it early, suggesting that researchers and administrators need to focus on a variety of areas. This research provides comprehensive knowledge about the development of scientific findings that will be evaluated over time.

## Introduction

Academic research has led to novel knowledge discoveries in various research areas and has contributed to human society’s prosperity. Generally, modern science focuses upon novelty which means that it is not uncommon for researchers to base their reseach on comparatively new papers. On the other hand, due to the nature of scientific findings, innovative discoveries are often accepted after evaluation and confirmation by researchers in related fields. This phenomenon is known as ‘delayed recognition’ (Garfield 1980, 1989, 1990), which is perceived as an example of unexpected discovery. New findings and theories are significantly crucial for scientific progress; however, initially, big findings are often restricted or neglected, as the scientific community is sceptical about them (Campanario 2009; Fang 2015).

Furthermore, information explosion prevents important ideas from penetrating the wall of established wisdom related to a subject, which could make more searches go unnoticed. Information explosion could cause an overflow in the publication of scientific research, which has resulted in 3–4% annual growth of science output in recent years (Petersen et al. 2019). Although this phenomenon has led to the prominence of article retrieval services like Google Scholars and researchers tend to cite previous but related articles (Sinatra et al. 2015), the high impact papers of recent years have mainly cited mainly new articles (Mukherjee et al. 2017). In other words, the significance and incluence of citing older papers is getting more important but still unclear.

**Fig. 1:**
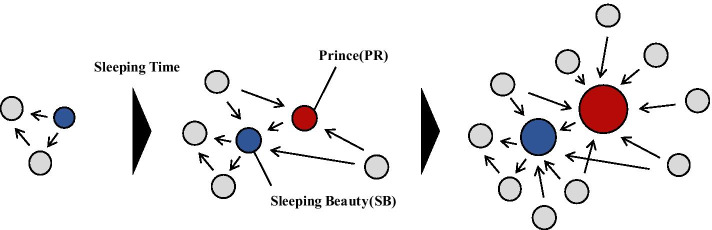
Overview of how a PR discovers an SB in the citation network. Each node represents papers and edges shows their citation. (1, left) As soon as an SB is submitted, it is indistinguishable from ordinary papers in the community without being cited much. (2, middle) When the PR realised the importance of the SB and cited it, it spread to the rest of the scientific community and led to the paper being co-cited. (3, right) A sub-field created by a pair of papers becomes widely recognised, and a new field can emerge from it

To observe delayed recognition from bibliometric data, the quantitative concept of delayed recognition is designated as the Sleeping Beauty (SB) phenomenon (Van Raan 2004). SB represents a set of papers that go uncited for a long time but are suddenly noticed after a particular time. In addition to the original definition of SB using depth of sleep (average citation), length of sleep (length of time with few citations), and awake intensity (degree of citation increase), several extended terms exist for the extraction of various cases of SB papers (Mazloumian et al. 2011; Bornmann et al. 2018). Initially, SB was regarded as a rare phenomenon in scientific progress, but recent research shows that it is far less exceptional than previously thought (Ke et al. 2015). Every SB has its own Prince (PR), which wakes the SB and introduces it to the broader research community by citing the SB document, as in Fig. 1. Classical cases of SB deal with being ahead of one’s time and the PR often helps to rediscover ’more ready’ SB papers (Van Raan 2004).

Many studies have positioned SBs and PRs in a specific field or category (Fazeli-Varzaneh et al. 2021; Hartley and Ho 2017; Ohba and Nakao 2012). Nevertheless, few systematic approach has been reported to date that can find the SB–PR pairs comprehensively from vast amount of articles. Miura explicitly examines the classification of the various types of scientific findings across respective scientific disciplines using SB and PR pairs in various fields (Miura et al. 2020). The research reveals that there are cultural differences between fields about how the PR discovers SB. However, some points in the study does not correspond to the actual situation of scientists. For example, it only discussed the field from the perspective of 2020, even though the field of science will change over time.

In this study, we quantitatively observe delayed recognition by obtaining SB–PR from a large-scale dataset, and clarify how PR discovers SB at the discovering time. We improved the extraction method of SB–PR pairs and the calculation method of rarity from a large-scale dataset to analyse delayed recognitions quantitatively. Moreover, we compared SB citations by PR with random citation and null models to show how predictable delayed recognition is.

This work is invited extension of the original presentation (Miura et al. 2020). In this paper, we have substantially extended our previous study by adding new contents to answer following question.

1. What type of papers have the methods proposed in the previous study (Miura et al. 2020) been able to obtain and how accurate it is?
2. What are the characteristics of citation from PR to SB compared to common citations and null models?
3. Is SB predictable when using the field at the time of the PR citation?

The rest of the paper is organised as follows. The following section describes this paper’s related works in terms of delayed recognition in various fields. Then, we provide the methods to extract a bulk of SB–PR pairs from a multi-disciplinary dataset and present the data. The following section defines citation rarity and methods to detect categories with an existing clustering algorithm. The next section reports the results of the analysis from the comprehensive Scopus dataset, and the section thereafter discusses them. The final section summarises the paper and suggests future research directions.

## Literature review

In the current section, we present the literature review on delayed recognition, SB, and PR.

### Delayed recognition

Delayed recognition has been studied for a long time to capture the life cycle of scientific findings with the development of bibliographic information data. Garfield was the first to present systematic knowledge of delayed recognition (Garfield 1980) and used the example of the beginning of radio astronomy by Karl Jansky and Mendel’s work on plant hybridisation (Garfield 1969). He stated that essential papers are overlooked because they are not linked to ‘well-known facts’. In other words, the mechanism of delayed recognition mainly depends on the prematurity of findings so that scientists cannot accept the result until the findings connect to common sense in the field (Stent 1972).

Delayed recognition can be compared to the bandwagon effect in research. The bandwagon effect is a phenomenon discussed in behavioural psychology: what is selected by the majority is more likely to be selected by others. It has been pointed out that the bandwagon effect exists in the citation of papers. For example, Asatani, in a task to estimate the closeness of each paper to the leading edge of research in the field based on the distributed representation of the citation network, found that the number of citations and the growth rate of keywords was higher for papers estimated to deal with topics closer to the leading edge of the field (Asatani et al. 2018). This finding suggests the existence of a bandwagon effect in which papers on trends in the field are more likely to be cited. Moreover, Sasaki found that the number of citations tends to be higher for papers with higher Pagerank (Page et al. 1999) in the paper citation network, suggesting that publishing papers that cite papers with strong influence leads to higher citations (Sasaki et al. 2020). As the number of citations of a paper is an important indicator of the contribution to the academic community and the evaluation of a researcher’s performance, the bandwagon effect suggests that researchers are more likely to increase their citations following highly cited papers. Delayed recognition has a complementary relationship with this bandwagon effect, such as Kuhn’s science revolution and normal science in research (Kuhn 1962), and may hold the key to unravelling the mechanism of knowledge production.

### Sleeping beauty

SB, a quantitative measure of delayed recognition, was proposed by Raan (Van Raan 2004), and many fields and structural features have been reported. In the innovation studies area, a variety of reasons are available to explain delayed recognition, such as the implementation of methods or tools and by increasing acceptance (Teixeira et al. 2017). Meanwhile, ophthalmological SBs are descriptions of new clinical diseases and innovation of medical and surgical treatments, which take time to confirm and extend the experience of new diseases (Ohba and Nakao 2012).

For the specific phenomenon of SB, Li focused on the phenomenon called ‘heartbeat’, which describes a paper cited once or twice a year before a sudden rise of citations (Li et al. 2014). In addition, some SB researchers have observed the phenomenon of ‘Spindle’, where a paper is highly cited in the first year or two after submission and then not cited at all, similar to the princess in a fairy tale who fell asleep after being pricked by the needle of a spinning wheel (Li and Ye 2012).

### Prince

The concept of the PR was proposed by Raan to trigger the awakeness of SB papers (Van Raan 2004). Typically, one PR corresponds to one SB, but there are several types of research which reports no PR–SB (Zong et al. 2018) or group PRs which trigger one SB (Van Raan 2015). There are several definitions to extract PRs, such as the first paper citing SB except self-citation (Braun et al. 2010; Van Raan 2015) and the most co-cited papers with SBs (Du and Wu 2017; Song et al. 2018). Which of these criteria to use depends on the type of delayed recognition to observe. For example, first citing PRs can reveal utterly unknown knowledge to scientists. Co-cited PR is appropriate for extracting articles that are closely related to SB. Compared to research on SBs, the discussion on PRs is continuous, and more detailed analysis is required.

For SBs, bibliometric research on the PRs has focused on a specific phenomenon or category of papers. Examining specifically the computer science category, it has been found that SBs contribute to some methods rather than its application, and their PRs have extended the model and methods established for SBs to make them applicable to other sub-fields (Dey et al. 2017). Braun also pointed out that PR tends to be submitted to journals with more than double the high impact factor of SB. This may indicate that it is possible for SB to be more visible to scientists in broader fields (Braun et al. 2010).

## Data

**Fig. 2:**
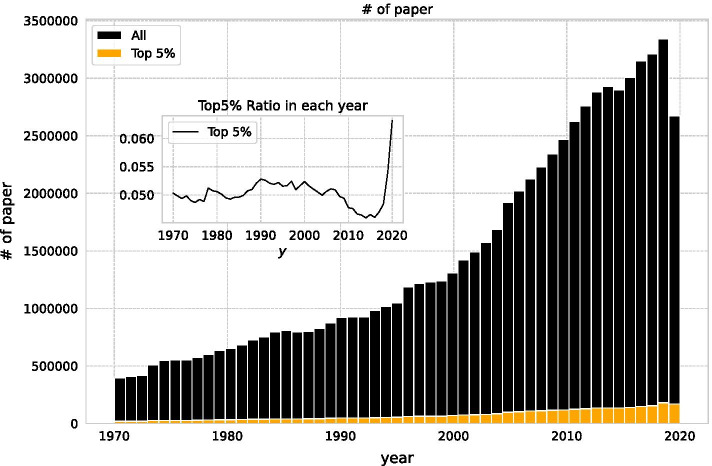
Publication year of papers. The black bar shows the whole number of papers published in the year. The orange bar represents the top5% in citation normalised by the average number of citations of papers submitted in the same field in the same year (Radicchi et al. 2008). The small graph shows the proportion of top papers to the total number of papers submitted in each year, which was stable at around 0.05 for papers in the period when there was sufficient time to obtain citations

We extracted comprehensive citation data from the Scopus Custom Dataset provided by Elsevier for all documents recorded in Scopus between January 1970 and September 2020. Scopus is the largest abstract and citation database of peer-reviewed literature and contains bibliometric metadata. Scopus data are available in bulk for research groups and used in other types of bibliometric research (Baas et al. 2020). The data consist of 73 million papers and 1.2 billion citations across almost all research fields (see Fig. 2). This dataset is large compared with other types of bibliometric research. As mentioned by Petersen, the number of papers has been growing every year (Petersen et al. 2019). Compared to year 2000, in 2019, 2.6 times more literature was submitted. There is a smaller value for 2020, as the data are limited to September. From this, we constructed an unweighted directed citation network and extracted the weak largest components. The network is composed of 61,527,485 nodes and 1,218,465,070 edges.

## Sleeping beauty and its prince extraction

This section provides the method to extract a large number of SBs and PRs from the multi-disciplinary dataset in Scopus.

### Sleeping beauty

There are various methods to identify SBs, such as average-based approaches (Van Raan 2004; Glänzel et al. 2003), which use average citations per year until each awakening year; quartile-based approaches (Costas et al. 2010), which focus on the citation ratio from its publication; and geometrical approaches, which use the angle or area of a yearly citation graph (Ye and Bornmann 2018). In this research, following the previous paper (Miura et al. 2020), we extract top 5% impactful papers from all the datasets and then use the ‘Beauty Coefficient’, which is a geometrical method proposed by Ke et al. (2015), to extract SBs.

Beauty Coefficient score *B* can be calculated as follows:

1 $$\begin{aligned} B = \sum _{t=0}^{t_m} \frac{\frac{c_{t_m}-{c_0}}{t_m}\cdot t + c_0 - c_t}{\max \{1, c_t\}} \end{aligned}$$

**Fig. 3:**
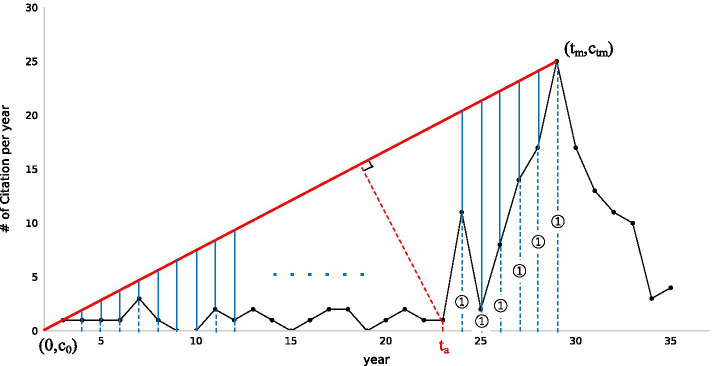
Illustration of the definition of the beauty coefficient *B* and its awakening year $t_a$. The black curve represents the number of citations $c_t$ received by the paper at age *t*. The awakening time $t_a \le t_m$ is defined as the age that maximises the distance from the line connceting points ($0, c_0$) and ($t_m, c_{t_m}$). *B* is calculated as a summation of the ratio of blue lines against each blue dot line from 0 to $t_m$

$c_t$ represents the number of citations that the paper received after its publication in the *t*th year, and $t_m$ represents the year in which the paper received maximum citations $c_{t_m}$. In other words, *B* is the sum of length scale between the straight line drawn from point $(0, c_0)$ to the point of maximum citation $(t_m, c_{t_m})$ and the citation curve, calculated for the number of citations in each year as shown in Fig. 3.

This method has two features. First, it is possible to achieve continuous score that draw a steeper citation curve with a stronger weight on the sleeping period of papers. In categorical classification methods such as average-based or quartile-based approach, SB is defined by a decision tree based on predefined criteria like “sleeping period equals to the span when the number of citations is less than *c*”. However, a simple threshold can easily lead to category bias since citation culture differs between disciplines. By using the Beauty Coefficient, it is possible to obtain the SBs with a particularly long sleep duration across multi-disciplinary dataset. Second, the Beauty Coefficient does not change even if the scales are all doubled, because this coefficient considers the shape of citation curves and does not observe the number of citations. In the case of mixed time and field data, there must be categorical citation bias (Ioannidis et al. 2019); hence, the Beauty Coefficient can ignore the scale and consider only the shape of the citation curve. To make the method robust to detect multi-disciplinary SB, a geometrical approach is appropriate for this time. Regarding the impact of the paper, we obtain papers with high citation counts, considering its chronological bias and field bias. In terms of the chronological bias, citation inflation has been reported in recent years with the increase in the number of papers, and it is possible that newer papers are more likely to be cited more often even if they have the same impact (Petersen et al. 2019). Moreover, as mentioned above, field bias is reported (e.g. physics, which is heavily cited, and mathematics, which is rarely cited). For this, we calculate the impact score normalised by the average number of citations of papers submitted in the same field in the same year because it is known that the number of citations in any field follows a universal distribution by this normalisation (Radicchi et al. 2008)

Figure 2 shows the year-wise distribution of top impact papers. With the number of papers increasing each year, the proportion of papers in the top 5% of impact has remained fairly constant over the period when sufficient citations can be obtained. Papers submitted in 2019 or 2020 still have mostly zero citations resulting in smaller average citation count, so some impactful papers in 2019 and 2020 are over-valued by normalisation.

Based on these criteria, we extract long-sleep and large-scale SBs satisfying

1. Normalised impact top 5% of papers
2. *B* top 1% of high impact papers extracted in 1

**Fig. 4:**
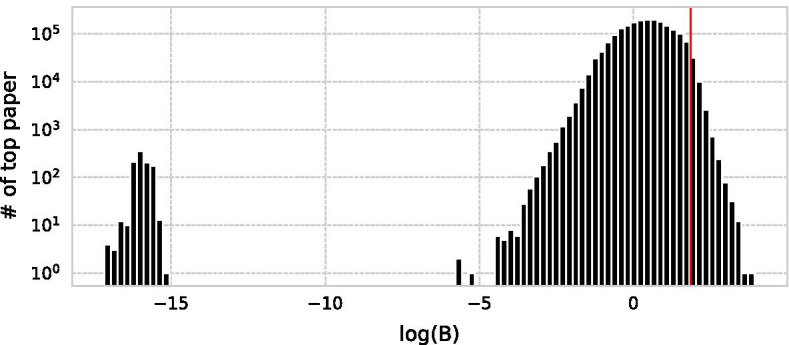
Distribution of *B* for top papers. A red dot line indicates the least score of SBs behind the peak of the majority of papers

For condition one, 5% is enough to obtain impactful paper because citation distribution is highly heterogeneous and less than 1% of papers have nearly half of its citation (Van Noorden et al. 2014). For condition two, Fig. 4 shows the distribution of *B* for the top 5% papers. Among the 3,684,785 top papers, 1,068,360 papers had *B* of less than 0 ($B<0$, the citation curve was convex upward), 632,729 papers had $B=0$ (the maximum citation in the year of submission or the year after submission), 987 papers had *B* close to 0 ($0<B<10^{-10}$, the citation rose almost linearly), and 1,982,709 papers had *B* well above 0 ($10^{-10}\le B$, the citation curve was convex downward). The top 1% of B, the line of $B=72.83$, is the part of the top paper that is above the peak and can be considered a paper with a sufficiently long sleep period. As a result, we extract 36,847 SBs from the entire dataset in Scopus.

**Fig. 5:**
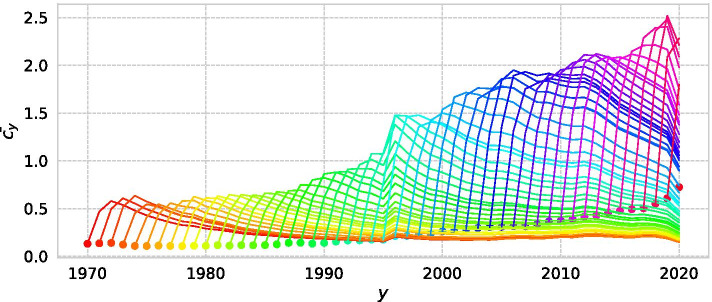
Distribution of citation in each year for all papers. Each coloured line refers to the average number of citation published in dotted point. Although citation inflation increases the citations to papers submitted in recent years, it does not increase the number of citations to older papers

When normalising citation, we must be concerned of the effect of citation inflation on the Beauty Coefficient. If older papers are more cited due to an increase in the number of papers, they will be over-cited even if they are not rediscovered, and only older papers could be extracted as SBs. After analysing the number of citations obtained in each year, we find that the chronological bias can be ignored in the calculation of *B* because older papers do not become more cited in recent years due to citation inflation (Fig. 5).

For each SB paper, an awakening year of SB paper $t_a$ is defined as the time at which $d_t$ takes the maximum value as follows:

2 $$\begin{aligned} t_a= argmax_{t\le t_m}\{d_t\} \end{aligned}$$

3 $$\begin{aligned} d_t= \frac{| (c_{t_m} - c_0)t - t_m c_t + t_m c_0|}{\sqrt{ (c_{t_m} - c_0)^2 + t_m^2}} \end{aligned}$$

$d_t$ is the length of the perpendicular line from point (*t*, $c_t$) down to the line joining (0, 0) and ($t_m$, $c_{t_m}$). The point where the length is the maximum is defined as the awakening year (Fig. 3).

**Fig. 6:**
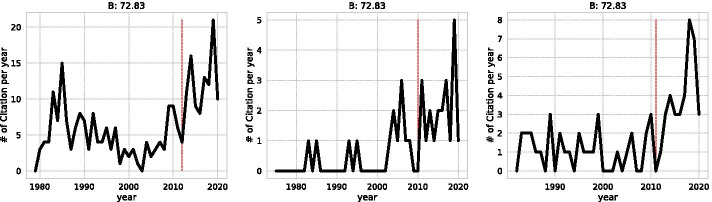
Yearly citation curve of the lowest B-score in SB papers. The dotted line indicates the year $t_a$ when the paper was discovered

In this study, we make a strong assumption that all papers with top 5% or more Beauty Coefficients are SB. To verify this assumption, we take the three papers with the lowest Beauty Coefficient and observe the annual change in the number of citations, as shown in Fig. 6. All of the papers experienced more than 30 years sleeping period, and the year with the lowest number of citations before the highest citation year is considered as $t_a$ for two out of three examples. This suggests that papers with the top 1% of Beauty Coefficient from the top 5% of citations have a high impact and a sudden increase in citations.

### Prince

Next, we propose a method to extract PR papers for each SB papers. ’The first report to cite SB’ is the original definition of a PR (Van Raan 2004). However, this definition is suitable only for cases of ‘coma sleep’, that is, cases wherein no attention is paid to SB (Van Raan 2015). With the improvement of a recommendation system of research papers, such as Google Scholar and Semantic Scholar, scientists can easily access minor but related articles. Furthermore, some SBs have a citation curve called second-act papers, which means that there is a period of time during which they were cited in addition to the period during which they were heavily cited. He et al. (2018). In such cases, the first paper cited does not necessarily cause the major citation peak of SB. As can be seen in Fig. 6, SB’s citation often peaks not after the first quote, but after decades. Therefore, as coma sleep occurs less often and there are second-act SBs, a co-citation criterion is a more appropriate method for finding a PR (Du and Wu 2017).

In this research, a PR is defined as the most highly co-cited paper published within 5 years of each SB’s awakening year. If paper $p_{PR}$ published in $t^{PR}$ is the PR of the paper SB, then $p_{PR}$ requires the following two conditions (4), (5):

4 $$\begin{aligned} t_{a_{SB}} - 5 \le t^{PR} \le t_{a_{SB}} + 5 \end{aligned}$$

5 $$\begin{aligned}&p_{PR} = argmax_{\hat{p}\in N_{SB}} (|\{e_{p^{\prime}, \hat{p}}|p^{\prime}\in N_{SB}\}|). \left ( e_{p^{\prime}, \hat{p}} = {\left\{ \begin{array}{ll} 1 &{} \text { (if } p^{\prime} \text { cites } \hat{p}) \\ 0 &{} \text{ (otherwise)} \end{array}\right. }\right) \end{aligned}$$

where $t_{a_{SB}}$ is the awakening year of SB and $N_{SB}$ is the set of papers which cites SB. Co-citing with SB can mean that research disseminated the SB findings to the related fields. In our method, we define one SB as corresponding to one PR. Although some SBs have been reported to have no PR in their papers, only 4 out of 126 SBs have no PR in previous studies (Zong et al. 2018). It has also been reported that some SBs are found in groups of multiple PRs (Van Raan 2015). In this study, we extract only papers that particularly triggered SB. As a result, 36, 847 SB–PR pairs are extracted from the dataset.

## The rarity of citations of delayed recognition

This section describes the methodology used to calculate the rarity of citations using clustering density to clarify whether the positioning of SB and PR in a citation network affect the predictability of SB.

### Definition of rarity

This subsection defines the SB–PR pair rarity concerning its citation probability between papers. There are various methods to calculate the citation probability between nodes, such as network embedding (Tang et al. 2015) and graph neural network (Zhang and Chen 2018), to predict the link probability. However, these methods are machinery expensive to calculate the citation rarity from billions of edges. Therefore, we utilise the category of papers to group the trends of citations. In bibliometrics, grouping with category is an effective method to capture the character of each paper (Ioannidis et al. 2019; Radicchi et al. 2008).

Citation probability is the density of edges between two categories in the PR publication year. When papers in a category comprising a PR paper cite the particular category that includes the SB paper, the presence of edges between SBs and PRs is not unusual. Hence, the density is high in this case. We define the density of pairs $d_{y, i, j}$ (citation from *i* to *j* in year *y*) as follows:

6 $$\begin{aligned} A^y_{i, j}= \sum _y A_{y, i, j}. \end{aligned}$$

7 $$\begin{aligned} d_{y, i, j}= \frac{A^y_{i, j}}{\sum _j A^y_{i, j} \cdot \frac{|c^y_j|}{N}} \end{aligned}$$

In the Eqs. (6) and (7), $A_{y, i, j}$ indicates the number of papers in the category, where *i* belongs to published during year *y* and cites the papers in the category *j* is in. Furthermore, $\sum _j A^y_{i, j} \cdot \frac{|c^y_j|}{N}$ represents the possible edges between *i*’s category and *j*’s category if all citation density is uniform, whereas $A^y_{i, j}$ showcases the actual edges between the two categories until year *y*. If $d_{y, i, j} > 1$, the density between *i*’s category and *j*’s category is higher than that in the uniform model, indicating that the connection is a common combination. The uniform model in this case refers to the edge density when the edge probability between clusters is held constant. In other words, it describes how dense an area is by comparing it with the case when the citation density between clusters is determined only by the number of nodes at that time. Using this method, we can measure fields with fewer citations even if they are cited by papers with different citation cultures (e.g. physics, which is heavily cited, and mathematics, which is rarely cited). Dividing the citation number by its category size enables us to obtain closer connections between smaller fields than between some of the larger ones. If a PR published in year $y_p$ from category $c_p$ cites an SB in category $c_s$, then the density of this SB–PR paper is described as $d_{y_p, c_p, c_s}$.

### Category extraction

There are two main methods for extracting a paper category: one is to use the labels given to the dataset and the other is to identify the field from keywords or citation. The former method is mainly used in bibliometrics, in which category labels assigned to journals are often used to focus on specific domains during the dataset extraction phase (Dey et al. 2017) and to discuss the results of the analysis obtained (Uzzi et al. 2013; Gates et al. 2019). However, externally assigned labels are rough; while they are useful for discussing macro trends, they are difficult to discuss at the micro-level of disciplines that a researcher can understand. For example, the All Science Journal Classification Codes label in Scopus, which classifies each journal into 334 categories, links hundreds of thousands of articles to a single label, but it is unlikely that all of them have the same topic. Meanwhile, topic extraction from keywords and citations is highly versatile as it allows granularity to be freely adjusted and is often used in the analysis of the overall research structure (Asatani et al. 2020). In particular, topic extraction using clustering of citation networks considers the connections between papers explicitly stated by the authors, even if they do not appear in the abstract and, thus, can be said to extract topics that match the perceptions of a researcher.

In this study, we use the Leiden method (Traag et al. 2019) to cluster the largest connected components of the citation network, and sub-clustering is performed until the size of the cluster with the largest number of nodes is less than 1% of the dataset. If the cluster size threshold is smaller, it is possible to obtain very small disciplines, but since modularity maximisation tends to be heterogeneous in size and results in a large number of disciplines with only a few papers, a maximum discipline size of about 1% of the total is appropriate. The Leiden algorithm is one of the dynamic hard clustering algorithms for optimising modularity *Q* defined as

8 $$\begin{aligned} Q = \frac{1}{2m} \sum _c \left ( e_c-\gamma \frac{K^2_c}{2m} \right) \end{aligned}$$

where $e_c$ is the actual number of edges in the community *c* and $\frac{K^2_c}{2m}$ is the expected number of edges, with $K_c$ being the sum of the degrees of the nodes in the community *c* and *m* the total number of edges in the network. Here, $\gamma$ denotes the resolution parameter (Fortunato and Barthelemy 2007) which controls the size of clusters. To extract many detailed categories, $\gamma$ is set to 1. The Leiden algorithm consists of three phases: (1) local moving of nodes, (2) refinement of the partition, and (3)aggregation of the network based on the refined partition. This enables the detection of communities faster than other normal clustering algorithms, such as Louvain methods (Blondel et al. 2008).

It should be noted that the Leiden method is not a time-series network clustering method. In other words, it represents the field from a certain point of time, but does not consider the temporal change of the field. For example, if research on silicon’s physical properties is discussed more frequently in the field of semiconductor application as the semiconductor field grows, the field of the input network might differ depending on the period of the network. Therefore, we calculate $d_{y, i, j}$ according to the year in which paper *i* was submitted. In particular, when paper *i*, which cites paper *j*, was published in year *y*, the density of citation from *i* to *j* is defined by the cluster calculated from the citation network up to year *y*. Given that cluster to which *i* belongs in year *y* is not necessarily the same as the cluster to which *i* belongs in year $y+1$, defining the field according to the year of the citing paper makes it possible to calculate the ‘subjective’ rarity at that time.

Through clustering, we first classify SB–PR pairs as in Table 1. Comparing the density at the time of discovery by PR and the cross-disciplinarity in 2020, it is possible to observe the feature of the field in which delayed recognition occurs and how it grows after discovery. Then, the density of citation $d_{y, i, j}$ is calculated for each citation according to the category in year *y*. This can clarify the rarity of the SB–PR citation compared with other citations even if both are inter-cluster citations.

**Table 1:** Classification of findings of sleeping by its Prince

Disciplinarity in 2020	Subjective rarity from the Prince at its own discovery time	
	Intra-disciplinary	Inter-disciplinary
Intra-disciplinary	Rediscovering findings and still integrated	Exploring findings and now integrated
Inter-disciplinary	Rediscovering findings and now separated	Exploring findings and still separated

**Fig. 7:**
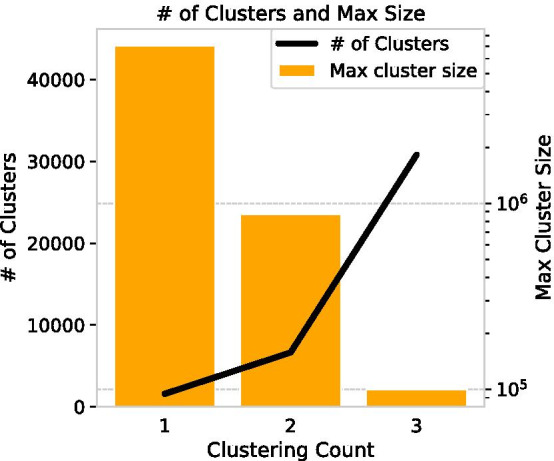
Sensitivity of clustering in 2020. Every sub-clustering, the maximum size of cluster is reduced by about 1/10, and the number of clusters is increased nearly tenfold

**Fig. 8:**
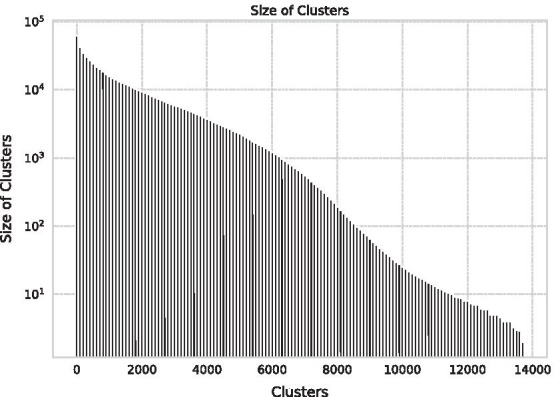
Cluster size in 2020. The cluster size increases on a log scale in the use of modularity maximisation

To determine what field each cluster represents in year *y*, 20 keywords are retrieved in order of frequency of the author keywords in the cluster to check consistency, and then 3 words are displayed. The results of the clustering in 2020 are shown in Figs. 7 and 8. Since 2020, the maximum cluster size was 99,512, less than 1% of the total, after sub-sub-clustering. Thus, these sub-sub-clusters should be regarded as fields in 2020. Clustering citation networks using modularity maximisation tends to result in some large clusters and many small clusters. However, in this case, we can obtain clusters even among disciplines which have a thin connection, and the heterogeneity of the cluster size is small as all clusters are divided twice.

## Results

This section provides the core result of large-scale SB–PR extractions and their features. First, we check the validity of the proposed method by comparing them to previous methods. Second, we compare the real citation network with the null model to statistically reveal the mechanism of delayed recognition. Third, we identify the categories which frequently feature delayed recognition. Fourth, we reveal the general features of delayed recognition. Finally, the relationship between Nobel Prize-winning papers and SB–PR pairs is presented.

### Validity of SB–PR pairs

**Fig. 9:**
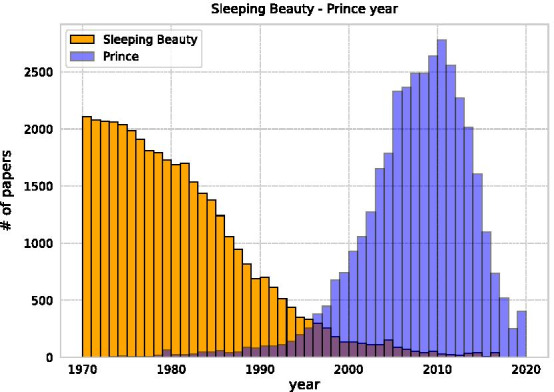
Publication year of SB and PR extracted in 2020. The orange bars show the distribution of submission years for SB, and the blue bars show the distribution of submission years for PR

**Fig. 10:**
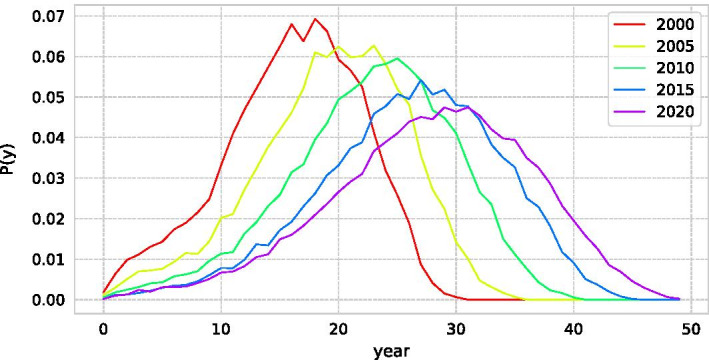
Probability of gap year between SB and PR. Length of sleep duration for SB and PR pairs in the dataset divided into 5-year increments. The mean values are 16.0, 19.3, 22.7, 25.9, and 27.9 years, which is about half of the total length of the dataset

Figure 9 presents the year-wise distribution of SB and PR. By definition, the greater the time distance between SB and PR, the larger the likely Beauty Coefficient. Therefore, most SBs are papers published between 1970 and 1990. For the same reason, PRs are published mostly between 2000 and 2015. The reason for the low PR rate of recent papers may be because not enough time has elapsed for each paper to gain sufficient citations. The gap year distribution reflects that SBs are typically discovered after around 27.9 years, which is almost half of the dataset length (Fig. 10). To illustrate the generality of this feature, we also compared the gap year of the SB–PR pairs that we were able to obtain by shortening the data in 5-year increments starting in 2020. In other words, the number of SB–PR pairs that can be obtained in a given data set is half the length of the entire data set.

We validate the proposed method by comparing the number of SB and PR pairs that are included in the pairs described in previous studies. The pairs of interest are 16 pairs of SB and PR, both of which are included in Scopus, out of the article pairs mentioned in previous research (Van Raan 2004, 2015; Dey et al. 2017; Du and Wu 2017; Song et al. 2018; Ohba and Nakao 2012; Teixeira et al. 2017). These gold standards might be a little small for validating the accuracy of result. However, there is still a debate about what type of paper SB is and whether PR exists, and only few papers extract SB and PR in pairs. In particular, as there is a possibility that some pairs which have not been pointed out in previous studies are actually delayed recognition, we focused our experiment on 16 pairs which are proved in previous studies. The future work is to ask experts in the history of science or researchers in various fields to create a gold standard.

To evaluate the validity of the SB extraction method, we compare the extraction accuracy of the three methods, including the proposed method, among 16 pairs of correct answers. The comparison method of the SB extraction is defined as follows:

- Average-based approach: The original and most ordinary method extracts the papers with more than 20 citations in 4 years after the discovery at which the average number of citations become less than 2 for more than 5 years after publication (Van Raan 2004).
- Quartile-based approach: The proportional methods extracts papers with less than half of the citations within 75% of the time after publication (Costas et al. 2010). The quartile-based method is adopted in this study for a comparison with the proposed method as well as the method with less parameters.

Next, we evaluate the accuracy of the proposed PR extraction method by comparing it with two other methods as follows:

- First citing paper: A method to obtain the paper which first cited SB (Van Raan 2004)
- Max co-citation and contribute to awakeness: A method to obtain the paper with the largest co-citation with SB and published within 5 years of the paper awakening (Miura et al. 2020). If there is a co-citation maximum paper within 5 years of $t_a$, the PR is extracted as in the proposed method. Conversely, if there is a co-citation maximum paper beyond 5 years, the pair itself is not counted as an SB–PR pair by this definition.

When assessing the accuracy of PR, we assume that any overlap in authors is correct even if the papers are not exactly the same, because researchers tend to submit many papers with similar topics of interest in a hot streak (Liu et al. 2018).

In terms of the accuracy of SB extraction, the proposed method succeeds in extracting 10 out of 16 SBs with a smaller number of proposals than the comparative method, which implies that the proposed method can obtain SBs with varying definitions (Table 2). Table 12 in the Appendix shows the detailed result of SB and PR extraction. The SB extracted by Ohba and Teixeira, which contains many examples of failed extraction, is not judged as SB because the original paper extracted SB based on the results measured in the limited datasets of ophthalmology and innovation engineering, respectively, and the Beauty Coefficient was not high compared with other fields. Moreover, the proposed PR extraction methods obtained 4 out of 10 PRs extracted in Table 12, which is better than other criteria (Table 3). The reasons that the extraction rate is not so high are discussed in the “Discussion” section.

**Table 2:** Accuracy of sleeping beauty

Method	Recall	Precision
Proposed method (top5% citation, top1% B)	10/16	10/36, 847
Avarage-based approach (Van Raan 2004)	8/16	8/100, 292
Quartile-based approach (Costas et al. 2010)	4/16	4/23, 427

**Table 3:** Accuracy of the Prince

Method	Accuracy
Proposed Method ($t_a$ 5 yearsMax Co-citation)	4/10
First Citing Paper (Van Raan 2004)	2/10
Max Co-citation and Contribute to Awakeness (Miura et al. 2020)	3/10

### Comparison with the null model

**Fig. 11:**
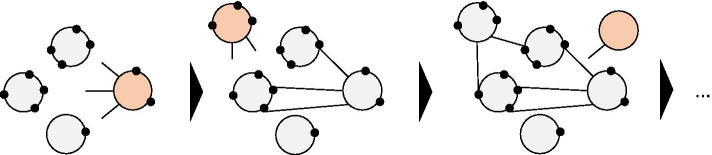
Illustration of the null model. Each node (paper) has a potential of $k_o$ (the number of black dot), which is defined by its 2020 citation, and every after adding new nodes every year, new edges are created with equal probability based on the remaining black dots

To statistically analyse the characteristics of the Beauty Coefficient in the empirical network, we investigated the specificity of SB in science by developing the following null model (Fig. 11). Assume that the number of citations in 2020 is the original potential of the paper, and create the recipients of the edges for each node. Each node (paper) has the recipients of the edges (citation). Under this potential parameter, we have added a new paper every year from 1970, and connect the edges with equal probability for the number of edge recipients i.e. the paper with the original potential $C_i$ (=citation) is evaluated immediately. Null model assumed that the potential of each paper can be detected immediately after citation. With this definition, the null model assumes that (1) the time series constraint is satisfied (no future papers are cited) and (2) the potential of each paper is measured by its 2020 citation (such that papers that have not been discovered yet are unknown).

**Fig. 12:**
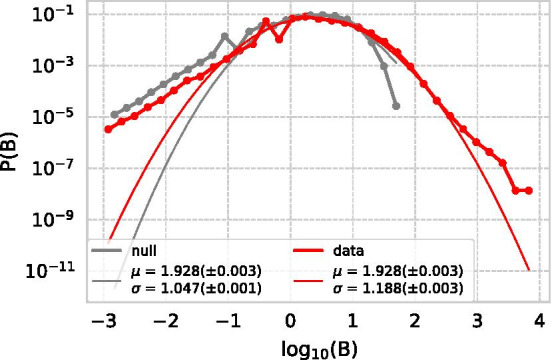
Distribution of *B* in the dataset and null model. The thin line is the result of log-normal fitting using the maximum likelihood method on each data

Compared with the null model, although the distribution of *B* was almost the same for most of the papers, empirical network was more fat-tailed (Fig. 12). Both the null model and the empirical data followed a log-normal distribution, with no dominant difference in their mean, but the empirical data had a larger variance. The results show that in science, it is indeed difficult to assess impact immediately, and that the deviation from the null model is significant, especially in areas where *B* is large.

### Field analysis

This subsection describes the relationship between delayed recognition and specific fields. Measured by category in 2020, the ‘COVID-19’ category contains the most PRs and SBs with a total of 179 (Table 4) and 158 (Table 5). The field of ‘COVID-19’ is the most typical example of delayed recognition, although it is a special case in that external factors caused a re-evaluation of the research on severe acute respiratory syndrome (SARS) and Middle East respiratory syndrome (MERS) in the early 2000s. The pair with the highest Beauty Coefficient in this field was SB of Netland, which examined the effects of SARS-Cov on the brainstem using Tg mice published (Netland et al. 2008), and PR of Li on COVID-19-induced dyspnea published, which has contributed to the core progress of COVID-19 research in the field (Li et al. 2020). The number of citations of the SB has risen from only 26 until 2019 to 306 now by large co-citation with the PR, which obtains 484 citations now. The proposed method identifies the fields where delayed recognition is likely to occur and retrieves pairs of papers to search for informative research to catch up with the history of the category.

It was found that delayed recognition is not confined to a particular field, but occur widely in many fields. Categories with a large number of SBs tend to have a large number of PRs. This suggests that fields with a large amount of delayed recognition are likely to form new academic fields, such as ‘2D materials’ or ‘aeroacoustics’. Focusing on the areas in which the number of SBs and PRs is asymmetric, the area concerning ’item response theory (IRT)’ in psychology has fewer PRs than SBs (104 SBs, 82 PRs). SBs in this category have influenced a wide range of fields, with a total of 38 different clusters of PRs, not only from the IRT field itself but also from other psychological categories related to ‘self-determination theory’ and ‘e-commerce’. The results enable us to quantify the categories in which delayed recognition is most likely to occur and how it spreads to other fields.

**Table 4:** Most frequent category including PR (category labelled by 2020)

Label	Keywords	# of PRs
Infectology (COVID-19)	(COVID-19, SARS-CoV-2, Coronavirus)	179
Philosophy (Ancient Rome)	(Aristotle, Plato, Rome)	131
Fluid Physics (Aeroacoustics)	(Aeroacoustics, Numerical simulation, Noise)	123
Material Chemistry (2D)	(MoS, 2D materials, transition metal dichalcogenides)	113
Neuroscience (Memory)	(Hippocampus, Memory, Episodic memory)	103
History (Spain)	(Slavery, Spain, History)	97
Geoscience (Liquefaction)	(Liquefaction, Sand, Anisotropy)	96
Geoscience (EOR)	(Enhanced oil recovery, Interfacial tension, Surfactant)	93
Sociology (AHP)	(AHP, TOPSIS, Decision making)	92
Physics (DFC)	(Electronic structure, Density functional theory, High pressure)	92

The table extended to the top 50 is presented in Table 13

**Table 5:** Most frequent category including SB (category labelled by 2020)

Label	Keywords	# of SBs
Infectology (COVID-19)	(COVID-19, SARS-CoV-2, Coronavirus)	158
Philosophy (Ancient Rome)	(Aristotle, Plato, Rome)	133
Fluid Physics (Aeroacoustics)	(Aeroacoustics, Numerical simulation, Noise)	133
History (Spain)	(Slavery, Spain, History)	113
Neuroscience (Memory)	(Hippocampus, Memory, Episodic memory)	112
Psychology (IRT)	(Item response theory, item response theory, Reliability)	104
Geoscience (EOR)	(Enhanced oil recovery, Interfacial tension, Surfactant)	101
Geoscience (Liquefaction)	(Liquefaction, Sand, Anisotropy)	98
Physics (DFC)	(Electronic structure, Density functional theory, High pressure)	97
Fluid Physics (Heat Transfer)	(MHD, Heat transfer, Nanofluid)	96

The table extended to the top 50 is presented in Table 14

### Density of citation of delayed recognition

**Fig. 13:**
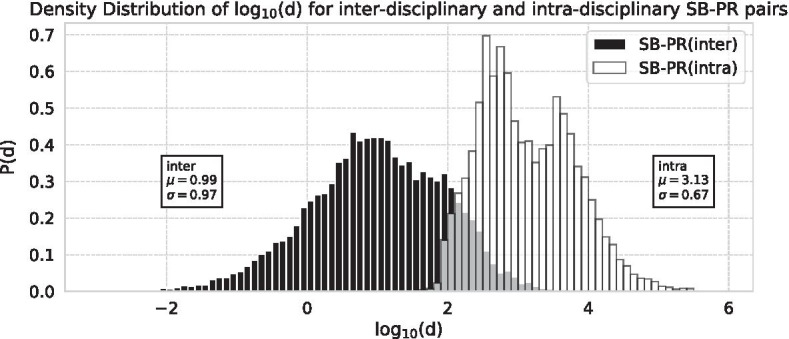
Density distribution of *d* for inter-disciplinary and intra-disciplinary at the time of their citation

We examine the influence of delayed recognition on disciplinary relationships using the disciplines at the time of the PR’s discovery and the disciplines in 2020. Figure 13 shows the distribution of $d_{y, c_p, c_s}$ for each type of citation point for the obtained SB–PR pairs. As the method uses modularity maximisation to identify categories, the mean of log$_{10} (d)$ for intra-disciplinary discoveries is 3.13, while the inter-disciplinary mean is 0.99. Around log$_{10} (d)$=2.5, there are both intra- and inter-disciplinary discoveries, meaning that the edges are about 300 times denser than those in the uniform model.

Table 6 shows the number of SB–PR pairs based on the cluster to which these papers belong compared with random edges. As a completely random sample would result in a large number of recent citations, we use the 36,847 random citations from edges to align to the year of publication of PR. We find that 75% of the pairs are still discussed as different fields in 2020 if they were discussed as various fields at the time of discovery and vice versa. Conversely, the rest of the 25% citations experience the dynamic change of category after PR discovered SB papers.

In particular, the number of citations that were inter-disciplinary at the time of PR submission (0.294) was lower than random (0.382), indicating that delayed recognition is likely to occur between the close sub-fields. Inconsistently, the dynamic category combination from inter-disciplinary citations at the time of PR was published to intra-disciplinary citations in 2020 (sbpr: 0.135, random: 0.096). It reveals that while most SB–PR pairs tend to occur in areas that can be called almost intra-disciplinary even at the time of citation, some discoveries have the ability to combine previously separate fields.

**Table 6:** Distribution of the sleeping beauty—Prince Pairs

Disciplinarity	Disciplinarity at discovering time		Total
in 2020	Intra-disciplinary	Inter-disciplinary	
Intra-disciplinary	sbpr:0.610 (random:0.460)	sbpr:0.153 (random:0.091)	sbpr:0.763 (random:0.551)
Inter-disciplinary	sbpr:0.096 (random:0.158)	sbpr:0.141 (random:0.292)	sbpr:0.237 (random:0.449)
Total	sbpr:0.706 (random:0.618)	sbpr:0.294 (random:0.382)	sbpr:1.000 (random:1.000)

**Fig. 14:**
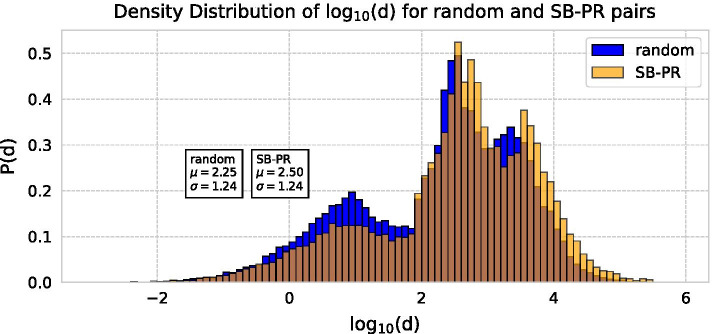
Density distribution of *d* for random pairs and SB–PR pairs

Based on this difference in distribution from random citation, we consider whether the timing of the submission of a PR is likely to predict whether the paper it cites is an SB or not. However, comparing the SB–PR pairs with random citation, no significant difference in distribution of *d* was found (Fig. 14). Although random citations tended to have a slightly higher *d*, both $\mu$ were within a $\sigma$ of each other. This implies that there was no deviation between the density of connection of categories and delayed recognition.

Therefore, we find that although SB–PR pairs are more likely to occur in intra-disciplinary citations, the density of the citation does not matter much if only it is intra-disciplinary. In other words, the relationship between delayed recognition and rarity does not increase linearly similar the possibility of overlooking and is not affected by the proximity of the fields.

### Relationship with Nobel Prize

To check the relationship between SB–PR pairs and other impact indicators, we examine 133 key papers related to Nobel Prize-winning findings selected by (Ioannidis et al. 2020) within the dataset. The analysis reveals that there are seven SBs and six PRs in Prize-winning papers. This shows that the Nobel Prize is awarded not only to SB, which is disruptive research rediscovered later, but also to a PR that discovers and disseminates previously dormant knowledge.

Table 7 shows the Nobel Prize winners and its publication year of SB. The SB papers that have been awarded the Nobel Prize have, on average, been evaluated after a sleep period of nearly 20 years and then awarded the Prize within a few years. The fact that there is a blank of several years, rather than immediately around the awakening year, suggests that the Nobel Prize is awarded when the field of research expands from SB and the Nobel Prize is later recognised as a major achievement. Thus, it is suggested that we may be able to obtain a large number of papers that form a field by exhaustively collecting SB. In addition, Table 8 shows how many Nobel Prize-winning papers are included in the PR instead of SB. Although the Nobel Prize is awarded for major contributions to science, such as those that break new ground, eight researchers were awarded the Nobel Prize in PR as well as SB. This shows that not only SB, who was the first to reveal hidden knowledge, but also PR, who spotted it, cited it, and spread its importance made a major scientific contribution.

**Table 7:** Prize winning authors of SB

Winning year	Author	Publish year	Awakening year
1997	Chu, Steven	1986	2001
2002	Brenner, Sydney	1974	1994
2007	Evans, Martin J.	1981	2000
2010	Suzuki, Akira	1979	2003
2010	Heck, Richard F.	1972	2002
2014	Hell, Stefan W.	1994	2007
2014	O’Keefe, John	1971	2002

**Table 8:** Prize winning authors of PR

Winning year	Author	Publish year	Sleeping beauty’s year
2000	Kandel, Eric R.	1982	1973
2002	Koshiba, Masatoshi	1998	1978
2009	Ramakrishnan, Venkatraman	2000	1974
2009	Greider, Carol W.	1990	1972
2015	Kajita, Takaaki	1998	1978
2017	Weiss, Rainer	2016	1974
2017	Barish, Barry C.	2016	1974
2017	Thorne, Kip S.	2016	1974

## Discussion

In this experiment, it is clear that the proposed method using the Beauty Coefficient can extract long-sleep and large-scale SB and it also covers previous findings accurately than the previous methods from a multi-disciplinary dataset. First, we discuss the relationship between the process of extraction method and its accuracy. Meanwhile, Van Raan’s method requires careful adjustment of the depth of sleep and awake intensity thresholds, given that the number of SB candidates increases as the data size increases and citations to existing papers are added as data increase. In fact, some of the papers that Van Raan himself once identified as SB were not identified as SB in the present dataset, even if using the same method. Therefore, Van Raan’s method is not sufficient to robustly identify SB across different datasets. Furthermore, Costas’ method extracts SB even if it is not a discontinuous finding because 75% of the time has passed since 2000, even if it is only a short period of sleep. The time elapsed from the submission changes as the time in the dataset increases so that a study which was an SB in the past might not be an SB in the new dataset, making it challenging to ensure the results’ reproducibility. By contrast, the Beauty Coefficient is calculated as the area bounded by the line connecting the point with the origin and the citation curve at the point with the highest number of papers per year. Hence, score *B* remains almost unchanged even if the number of citations in years other than the year of maximum citation were to change several times. As the Beauty Coefficient can ignore the number of citations after the peak of the citation curve, it is considered able to extract SB with high relevance by simply applying SB after adding a new paper.

Next, we consider the PR extraction described in Table 3. Out of 10 SBs, only 4 PRs, consistent with the results of previous studies, can be extracted using the proposed method. This is probably because the proposed method focuses only on ‘field formation’ based on co-citation among the many PR criteria. Table 9 summarises the criteria of the PR and its interpretation. The 10 PRs used in this evaluation are extracted by combining definitions 1–4 in previous studies. However, when only definitions 3 and 4 are used as in the proposed method, the replication rate of the previous studies is not high. This suggests that the three successful PRs are ‘discovered’, ‘re-evaluated’, and ‘fielded’ in one paper, while the seven unsuccessful PRs are discovered, re-evaluated, and fielded in another paper.

**Table 9:** Prince criteria and interpretation

	Prince criteria	Interpretation of the criteria
Definition 1	First paper citing SB	Discovering SB
Definition 2	High Citation	Reevaluating SB
Definition 3	High Co-citation with SB	Field Formation from SB
Definition 4	Published around SB awakening year	Contribute to SB spreading

The density of citation $d_{y, i, j}$ provides quite an interesting implication for delayed recognition. This study shows that delayed recognition occurs more often within categories. In case PR cites inter-disciplinary SB, it tends to later combine and form fields. For instance, green fluorescent protein (GFP) which was the subject of a Nobel Prize awarded in chemistry in 2006 on the colouration mechanism of the Aequorea, was a representative case of strong relation ($d=7952.7$) before the findings. The SB had been discussed for a long time in a limited field, and its potential was expanded by using genetic engineering techniques. The isolation of GFP from the Aequorea by Shimomura et al. (1962) was reported in the 1960s, but it did not attract much attention from other fields owing to the difficulty of luminescence in other animals and plants. However, in the 1990s, Chalfie et al. (1994) and Heim (1995) found a way to use GFP as a marker protein in genetic engineering, contributing significantly to the field’s growth. Finally, in 1996, the publication of Cormack’s work on enhancing luminescence has contributed to the further expansion of genetic engineering. In our method, the flow of this discovery can be quantitatively obtained in the form of SB by Morise et al. (1974) and PR by Cormack et al. (1996).

Regarding a rare combination with other fields, Schmidhuber’s work on deep learning survey (Schmidhuber 2015) caused exploring findings to awaken multiple SBs (18 SBs; 12 SBs are d [MYLT] 10). The PR was a comprehensive survey paper published in 2015, only before deep learning became an active topic of discussion in the ML domain, and provided the basis for deep learning to start being used in various domains, including speech, language, and image. Consequently, it awoke SBs in a total of nine fields, including arts and humanities and psychology, as well as computer science.

Tables 10 and 11 present the pairs with the highest and lowest density of citations, respectively. Using the proposed method, delayed recognition across multiple fields can be obtained as a pair of papers from a citation network. This study quantitatively shows that there are both types of SB–PR findings in cross-disciplinary delayed recognitions.

**Table 10:** Top 5 SB–PR pairs with the highest d

Year	Title	d
SB:2013	SB: Algorithm for automaton specification for exploring dynamic labyrinths	$2.9 \times 10^5$
PR:2017	PR: Construction of cellular automata over hexagonal and triangular tessellations for path planning of multi-robots	
SB:1973	SB: Digital pachydermia of the first phalanges from dermic connective tissue hyperplasia and hypodermic aplasia	$2.8 \times 10^5$
PR:2014	PR: Pachydermodactyly successfully treated with triamcinolone injections	
SB:1981	SB: The theory of uranium enrichment by the gas centrifuge	$2.8 \times 10^5$
PR:2011	PR: The generalized Onsager model for the secondary flow in a high-speed rotating cylinder	
SB:1980	SB: Onsager’s pancake approximation for the fluid dynamics of a gas centrifuge	$2.8 \times 10^5$
PR:2011	PR: The generalized Onsager model for the secondary flow in a high-speed rotating cylinder	
SB:2016	SB: Imposing patient data privacy in wireless medical sensor networks through homomorphic cryptosystems	$2.7 \times 10^5$
PR:2019	PR: A methodology for the analysis of consistent hashing

**Table 11:** Top 5 SB–PR pairs with the lowest d

Year	Title	d
SB:1972	SB: A double sampling scheme for estimating from misclassified multinomial data with applications to sampling inspection	$6.1 \times 10^{-4}$
PR:2004	PR: Increasing power for tests of genetic association in the presence of phenotype and/or genotype error by use of double-sampling	
SB:1977	SB: Evolution and tinkering	$1.5 \times 10^{-3}$
PR:1999	PR: From molecular to modular cell biology	
SB:1993	SB: Principal components analysis (PCA)	$1.8 \times 10^{-3}$
PR:2012	PR: Potential risk for healthy siblings to develop schizophrenia: Evidence from pattern classification with whole-brain connectivity	
SB:1976	SB: Masks for Hadamard transform optics, and weighing designs	$3.0 \times 10^{-3}$
PR:2012	PR: On a conjecture concerning the Frobenius norm of matrices	
SB:1975	SB: Microstructural control in lead alloys for storage battery application	$3.8 \times 10^{-3}$
PR:1998	PR: Structure, mechanical properties and electrical resistivity of rapidly solidified Pb–Sn–Cd and Pb–Bi–Sn–Cd alloys	

However it does not depend on the density of connection between the pairs. Table 6 and Fig. 14 show that both SB–PR citations and random citations are dominated by citations within the field, so delayed recognition cannot be attributed to the occurrence of previously unseen combinations. In bibliometrics, novelty of research is usually calculated by atypical combinations, measured by using combinations of new journals and co-citations. Delayed recognition is not the result of such a combination, but the re-evaluation of the papers already around is considered to be a major key factor.

The relationship between the Nobel Prize and delayed recognition seems to be closely related to the award process by the Nobel Committee. The Nobel Prize is often awarded to the person who has made the most significant contribution to science development, based on an exhaustive search of the candidate’s research, including surrounding fields. The fact that SB and PR are among the many studies that have been investigated by humans and contributed to the development of science suggests that delayed recognition plays a vital role in the process of scientific evolution.

## Conclusion

In this paper, we proposed a method to retrieve long-sleep and large-scale SB–PR pairs, a particular type of delayed recognition, from the entire Scopus dataset. This paper aimed to clarify how PR discovers SB at the discovering time in order to discover insights on the development of science. We use the number of citations and the Beauty Coefficient to obtain reasonable pairs from a large dataset spanning several fields, which is more accurate than existing methods. Compared with the null model wherein the potential of a paper is detected and cited immediately after its submission, the Beauty Coefficient of the real citation network is fat-tailed. This implies that the papers causing the delayed recognition are scattered heterogeneously. Meanwhile, the predictability of SB and PR could not be determined simply by the field in which the paper was submitted. Moreover, citation of SBs by PRs occurs within the same field of proximity as random citation. Therefore, the quantitative understanding of the delayed recognition can be deepened through research to identify the potential of SB as much as possible in advance. Moreover, compared with Nobel Prize-winning papers, in the dataset used in this study, not only SBs but also PRs were awarded, implying that both SBs and PRs contribute to the development of science. This research quantitatively proved these findings using a data-based approach.

This study provided a method to discuss the development of science quantitatively in terms of delayed recognition. However, there are clearly certain limitations in using the same paradigm for all science fields, and the structure of scientific discoveries that lie beneath individual fields should be included in the discussion. For future work, by looking not only at the main characters, namely, SB and PR, but also at supporting characters, it would be possible to observe how previously overlooked discoveries can be extended.

## Data Availability

The datasets analysed during the current study are available in the Scopus Custom Dataset provided by Elsevier if ordered.

## Appendix

See Tables 12, 13 and 14.

**Table 12:** Validation results of SB–PR pairs

Mentioned	Pairs	Result
Van Raan (2004)	SB: Massive N = 2a supergravity in ten dimensions (1986) PR: Dirichlet Branes and Ramond-Ramond Charges (1995) Extracted PR: Duality of type-II 7-branes and 8-branes (1996)	SB:OK PR:×
Van Raan (2015)	SB: Gauge singlet scalars as cold dark-matter (1994) PR: The Minimal Model of nonbaryonic dark matter: a singlet scalar (2001) Extracted PR: The minimal model of nonbaryonic dark matter: A singlet scalar (2001)	SB:OK PR:OK
Van Raan (2015)	SB: Theoretical possibility of stage corrugation in Si and Ge analogs of graphite (1994) PR: Two- and one-dimensional honeycomb structures of silicon and germanium (2009) Extracted PR: Two- and one-dimensional honeycomb structures of silicon and germanium (2009)	SB:OK PR:OK
Dey et al. (2017)	SB: Rough Sets (1982) PR: A Generalized definition of rough approximations based on similarity (2000) Extracted PR:Rough sets theory for multicriteria decision analysis (2001)	SB:OK PR:OK
Dey et al. (2017)	SB: The viterbi algorithm (1973) PR: A tutorial on hidden Markov models and selected applications in speech recognition (1990)	SB:×
Du and Wu (2017)	SB: Breaking the diffraction resolution limit by stimulated-emission: Stimulated-emission-deplesion fluorescence micorscopy (1994) PR: Imaging intracellular fluorescent proteins at nanometer resolution (2006) Extracted PR: Far-field optical nanoscopy (2007)	SB:OK PR:×
Du and Wu (2017)	SB: Breaking the diffraction resolution limit by stimulated-emission: Stimulated-emission-deplesion fluorescence micorscopy (1994) PR: Sub-diffraction-limit imaging by stochastic optical reconstruction micorscopy (STORM) (2006) Extracted PR: Far-field optical nanoscopy	SB:OK PR:×
Song et al. (2018)	SB: Exact stochastic simulation of coupled chemical reactions (1977) PR: Efficient exact stochastic simulation of chemical systems with many species and many channels (2000) Extracted PR: Efficient exact stochastic simulation of chemical systems with many species and many channels (2000)	SB:OK PR:OK
Ohba and Nakao (2012)	SB: Pharmacologic weakening of extraocular muscles (1973) PR: Botulinum Toxin Injection into Extraocular Muscles as an Alternative to Strabismus Surgery (1980) Extracted PR: A multicenter, double-blind, randomized, placebo-controlled study of the efficacy and safety of botulinum toxin type A in the treatment of glabellar lines (2002)	SB:OK PR:×
Ohba and Nakao (2012)	SB: APPLANATION TONOMETRY AND CENTRAL CORNEAL THICKNESS (1975) PR: Idiopathic polypoidal choroidal vasculopathy (IPCV) (1990) Extracted PR: Human corneal thickness and its impact on intraocular pressure measures: A review and meta-analysis approach (2000)	SB:OK PR:×
Ohba and Nakao (2012)	SB: Increased Corneal Thickness Simulating Elevated Intraocular Pressure (1978) PR: Idiopathic polypoidal choroidal vasculopathy (IPCV) (1990) Extracted PR: Human corneal thickness and its impact on intraocular pressure measures: A review and meta-analysis approach (2000)	SB:OK PR:×
Ohba and Nakao (2012)	SB: Blindness caused by photoreceptor degeneration as a remote effect of cancer (1976) PR: Photoreceptor Degeneration: Possible Autoimmune Disorder (1983)	SB:×
Ohba and Nakao (2012)	SB: Ocular perforation following retrobulbar anesthesia for retinal detachment surgery (1978) PR: Current concepts in retrobulbar anesthesia (1985)	SB:×
Ohba and Nakao (2012)	SB: Sources of error with use of Goldmann-type tonometers (1993) PR: The expanding clinical spectrum of idiopathic polypoidal choroidal vasculopathy (1997)	SB:×
Teixeira et al. (2017)	SB: The balanced scorecard–measures that drive performance. (1992) PR: The balanced scorecard: A foundation for the strategic management of information systems (1999)	SB:×
Teixeira et al. (2017)	SB: From value chain to value constellation: designing interactive strategy. (1993) PR: Evolving to a New Dominant Logic for Marketing (2004)	SB:×

× in SB means that the method does not regard the paper as SB. × in PR means that the method extract other papers as PR

**Table 13:** Most frequent category including PR (Category labelled by 2020)

Keywords	# of PRs
(COVID-19, SARS-CoV-2, Coronavirus)	179
(Aristotle, Plato, Rome)	131
(Aeroacoustics, Numerical simulation, Noise)	123
(MoS, 2D materials, transition metal dichalcogenides)	113
(Hippocampus, Memory, Episodic memory)	103
(Slavery, Spain, History)	97
(Liquefaction, Sand, Anisotropy)	96
(Enhanced oil recovery, Interfacial tension, Surfactant)	93
(AHP, TOPSIS, Decision making)	92
(Electronic structure, Density functional theory, High pressure)	92
(MHD, Heat transfer, Nanofluid)	89
(Truth, Explanation, Metaphysics)	83
(Item response theory, item response theory, Reliability)	82
(Microstructure, Retained austenite, Mechanical properties)	79
(Distributed video coding, Distributed source coding, Multiple description coding)	78
(Rock mechanics, Acoustic emission, Numerical simulation)	76
(Internal waves, Climate change, Sea level)	74
(Medicinal plants, Ethnobotany, Antioxidant)	73
(Kalman filter, Target tracking, Particle filter)	69
(Psychoanalysis, Trauma, Countertransference)	67
(Metacognition, Learning, Problem solving)	66
(Demand response, Smart grid, Optimization)	66
(Rats, pigeons, Rat)	65
(Precipitation, Rainfall, Remote sensing)	64
(Shale gas, Shale, Ordos Basin)	64
(Concrete, Compressive strength, Size effect)	63
(Motor control, Motor learning, Human)	63
(Heavy oil, Enhanced oil recovery, SAGD)	63
(Power system, Voltage stability, FACTS)	62
(Sequence stratigraphy, Holocene, Sediment transport)	62
(Motion, Visual cortex, Vision)	61
(Hydraulic fracturing, Hydraulic fracture, Numerical simulation)	61
(Shakespeare, Gender, Reformation)	61
(Heat transfer, Flow boiling, Boiling)	60
(Silicon, Solar cell, Nanowires)	59
(Density functional theory, DFT, density functional theory)	59
(Polyphasic taxonomy, Taxonomy, 16S rRNA gene)	58
(Black holes, Black Holes, Classical Theories of Gravity)	57
(Influenza, Influenza virus, Avian influenza)	56
(Elections, Political parties, European Union)	56
(Prejudice, Attitudes, Social identity)	56
(Dielectric properties, Ceramics, Ferroelectrics)	56
(Thermoelasticity, Generalized thermoelasticity, Laplace transform)	56
(Microstructure, Mechanical properties, Martensitic transformation)	55
(Spintronics, Magnetic tunnel junction, MRAM)	54
(Social media, Advertising, Consumer behaviour)	53
(Finite element method, Finite elements, Fluid-structure interaction)	53
(Complex networks, Complex network, Social networks)	53
(Pile, Piles, Pile foundation)	53
(Water splitting, Photocatalysis, water splitting)	53

**Table 14:** Most frequent category including SB (Category labelled by 2020)

Keywords	# of SBs
(COVID-19, SARS-CoV-2, Coronavirus)	158
(Aristotle, Plato, Rome)	133
(Aeroacoustics, Numerical simulation, Noise)	133
(Slavery, Spain, History)	113
(Hippocampus, Memory, Episodic memory)	112
(Item response theory, item response theory, Reliability)	104
(Enhanced oil recovery, Interfacial tension, Surfactant)	101
(Liquefaction, Sand, Anisotropy)	98
(Electronic structure, Density functional theory, High pressure)	97
(MHD, Heat transfer, Nanofluid)	96
(MoS, 2D materials, transition metal dichalcogenides)	94
(Truth, Explanation, Metaphysics)	87
(AHP, TOPSIS, Decision making)	83
(Microstructure, Retained austenite, Mechanical properties)	83
(Internal waves, Climate change, Sea level)	82
(Kalman filter, Target tracking, Particle filter)	81
(Medicinal plants, Ethnobotany, Antioxidant)	80
(Distributed video coding, Distributed source coding, Multiple description coding)	80
(Psychoanalysis, Trauma, Countertransference)	78
(Rock mechanics, Acoustic emission, Numerical simulation)	71
(Metacognition, Learning, Problem solving)	70
(Heavy oil, Enhanced oil recovery, SAGD)	69
(Motion, Visual cortex, Vision)	69
(Precipitation, Rainfall, Remote sensing)	68
(Power system, Voltage stability, FACTS)	66
(Sequence stratigraphy, Holocene, Sediment transport)	64
(Turbulence, Large eddy simulation, Turbulent flow)	64
(Rats, pigeons, Rat)	64
(Polyphasic taxonomy, Taxonomy, 16S rRNA gene)	61
(Hydraulic fracturing, Hydraulic fracture, Numerical simulation)	61
(Concrete, Compressive strength, Size effect)	60
(Black holes, Black Holes, Classical Theories of Gravity)	60
(Prejudice, Attitudes, Social identity)	60
(Microstructure, Mechanical properties, Martensitic transformation)	60
(Density functional theory, DFT, density functional theory)	59
(Silicon, Solar cell, Nanowires)	59
(Composites, Delamination, Composite)	58
(Motivation, Self-efficacy, Self-determination theory)	58
(Heat transfer, Flow boiling, Boiling)	58
(European Union, EU, European integration)	57
(Luminescence, Photoluminescence, Phosphor)	56
(Demand response, Smart grid, Optimization)	55
(Pile, Piles, Pile foundation)	55
(Fatigue, Fatigue crack growth, Fatigue life)	55
(Survival analysis, Missing data, Clinical trials)	54
(Elections, Political parties, European Union)	53
(Bullying, Adolescence, Parenting)	53
(Archaeology, Neolithic, Bronze Age)	52
(Water splitting, Photocatalysis, water splitting)	52
(Linear systems, Model reduction, Robust control)	52

## Abbreviations

SB: Sleeping beauty
PR: Prince
CDF: Cumulative distribution function
